# Translation, Cross-Cultural Adaptation to Malay, and psychometric evaluation of the AIM-IAM-FIM questionnaire: Measuring the implementation outcome of a community-based intervention programme

**DOI:** 10.1371/journal.pone.0294238

**Published:** 2023-11-16

**Authors:** Hazeqa Salleh, Richard Avoi, Haryati Abdul Karim, Suhaila Osman, Nirmal Kaur, Prabakaran Dhanaraj

**Affiliations:** 1 Faculty of Medicine & Health Sciences, Department of Public Health Medicine, University Malaysia Sabah, Sabah, Malaysia, Malaysia; 2 Sabah State Health Department, Ministry of Health Malaysia, Sabah, Malaysia; 3 Kota Kinabalu District Health Office, Ministry of Health Malaysia, Sabah, Malaysia; School of Nursing Sao Joao de Deus, Evora University, PORTUGAL

## Abstract

**Background:**

The implementation outcomes determine the success and progress of a community-based intervention programme. The community is an important stakeholder whose effects should be assessed. Nevertheless, Malaysia has limited instruments for determining outcome measurements. This research aimed to develop Malay versions of the Acceptability, Appropriateness, and Feasibility Intervention Measures (AIM-IAM-FIM) questionnaire, which evaluates the implementation outcome of the programme.

**Methods:**

A methodological study of the translation and validation of the implementation outcome measures was conducted from March 2022 until December 2022. Three key analyses were conducted: (1) translation and validation; (2) factor investigation and extraction (n = 170); and (3) scale evaluation (n = 235).

**Result:**

The Malay version measuring the implementation outcome measures of a community-based intervention programme was produced after extensive translation and modification, and it consisted of a single dimension with seven items. The content validity index was 0.9, the exploratory factor analysis showed that the KMO measure of sample adequacy was 0.9277, and Bartlett’s sphericity test was statistically significant. Cronbach’s alpha was good, with a level of 0.938. The single factor structure fitted the data satisfactorily [χ2 (p-value of 0.002), SRMR = 0.030, CFI = 0.999, RMSEA = 0.079, TLI = 0.998]. Factor loading for all items was > 0.7.

**Conclusion:**

The 7-item Malay version of the AIM-IAM-FIM survey instrument is valid and reliable for assessing the acceptability of a community-based intervention study and is applicable to other fields. Future studies in psychometric evaluation are recommended in other states due to the variety of Malay dialects spoken across Asia. The scale may also benefit other areas where the language is spoken.

## Introduction

Community-based interventions are purposeful actions taken to improve the targeted needs of people in a defined local community [1]. In public health, such intervention are often community-focused, and used population-based strategies to influence the norms, practices, attitudes, perceptions, habits, and behaviours of all or a certain group of people [2]. After all, involvement of the community itself in an intervention is important since according to the Ottawa Charter, empowering the community allowed them to accept and be more involved in the intervention initiated [3,4].

Globally, research has been done on community-based intervention in various fields, including health. Several community-based health programmes also have been implemented in Malaysia, targeting significant health issues, such as *Komuniti Sihat Perkasa Negara* (KOSPEN) [5,6], Communication for Behavioural Impact (COMBI) program [7], MyChampion [8], and others. Several recommended models for community-based interventions include: (i) the community as the context for measures; (ii) the community as the target of change; (iii) the community as a commodity; and (iv) the community as the operator [9]. These programmes aim to empower the community through community participation, improve health, and improve the community’s ability to deal with health issues.

Therefore, a community-based programme should be evaluated to see if it produced the intended outcome to ensure sustainability. In an implementation research, the outcomes are the implementation outcomes (acceptability, appropriateness, and feasibility); service outcomes (efficiency, effectiveness, and timeliness); and client outcomes (satisfaction) [10]. However, due to the complexity of health care, recommendations were given to consider other indicators that consider different dimensions of implementation result qualities [11], like the usage of implementation strength (intensity, degree, quality) [12].

However, a lack of accurate and validated tools in Malay may explain why there has been so little progress in evaluating community-based interventions in the country. Most of the available studies that have been done in this country include the quantitative and qualitative assessment of awareness, knowledge, and perception, as well as the effectiveness of the community-based health programme assessed through service outcome indicators [6,7]. Various studies have been conducted in countries that employ implementation outcome measures to assess services or programmes [13,14]. Nevertheless, due to cultural and healthcare system variations, implementation result studies from other nations cannot be generalised to Malaysia.

Therefore, the focus of this study was to create a Malay version of the AIM-IAM-FIM questionnaire, originally created by Weiner et al. [15], which evaluates the indicators of success in programme implementation, which were acceptability, appropriateness, and feasibility factors. This questionnaire was then used on two different community based programmes with similar operational concept where they used community volunteers to run their projects which were Komuniti Sihat Perkasa Negara (KOSPEN) programme, and the Measles Intervention Involving Community Action (MIICA) programme. The KOSPEN program involved the preventive activities to address the non-communicable diseases while the MIICA programme is an initiative to promote measles immunisation uptake. Ultimately, having valid tool to evaluate the implementation outcome of these programmes could enhanced their quality and effectiveness.

## Methods

### Design

**Fig 1:**
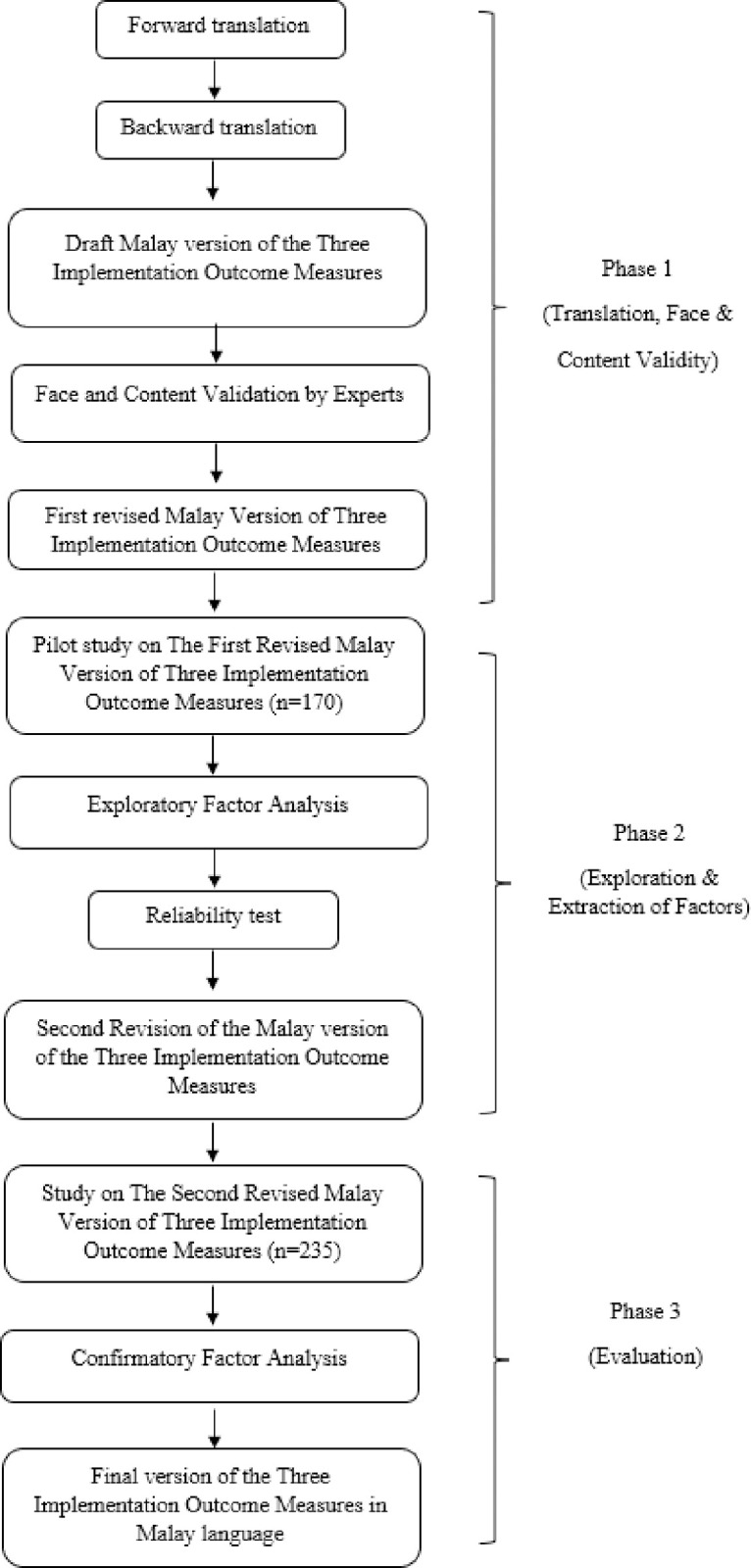
Overview of the research processes involved in creating the Malay version of the AIM-IAM-FIM questionnaire.

A methodological study with three phases: (1) translation and validation; (2) factor investigation and extraction (n = 170); and (3) scale evaluation (n = 235) was done per standards for creating and testing scales [16]. **Fig 1** shows the translation, exploration, extraction, and evaluation processes.

### AIM-IAM-FIM questionnaire overview

The implementation outcome measures an intervention’s acceptability, appropriateness, and feasibility [15]. Each construct has four parts, and the type of answer depends on the 5-point Likert scale: 1, completely disagree; 2, disagree; 3, neither agree nor disagree; 4, agree; and 5, completely agree. In the English version, the 12-item questionnaire took less than 15 minutes on average. Adding the scores for each item in each component yielded the overall score. The intervention is more likely to be accepted, appropriate, and feasible with a higher score. The source for this questionnaire is freely available to the public [15]. A request for translation purposes was asked from the owner of the original questionnaire, and permission for the purposes was granted.

### Translation

The three implementation outcome measures were translated into Malay based on the International Society for Pharmacoeconomics and Outcomes Research (ISPOR) Guideline for Translation and Cultural Adaptation Process [17] and the Guidelines for the Process of Cross-Cultural Adaptation of Self-Report Measures [18]. Four translators participated in this research (two performed the forward translation, and another two performed the backward translation). Two separate translators worked together to develop a unified translation report after completing the forward translation procedure (Malay to English). Two more translators were given the translated questionnaire to perform a reverse translation (Malay to English). They were both native Malay speakers with a working knowledge of English. They eventually collaborated on a single document. The final Malay version of the three implementation outcome measures was obtained after the study team compared, confirmed, and corrected the two translations.

### Face and content validation

Nine experts evaluated the validity of the Malay version of the AIM-IAM-FIM questionnaire. The expert panel includes five public health specialists, two family health physicians, and two health officers managing community-based programmes and health promotion in the district. Each item was analysed separately to see whether or not it met the criteria for content validity, which includes checking for the following: a) confirmation that the translated instrument measures what it is intended to measure; b) suitability; and c) congruence with the aims of the study. The experts were then asked to assess the items’ face validity, considering factors like readability, coherence, layout, complexity, item classification, answer simplicity, language formation, and phrasing. The experts rated the content validity using a four-point Likert scale: 1 = not relevant; 2 = somewhat relevant; 3 = quite relevant; and 4 = highly relevant. Face validity was also rated using a four-point Likert scale: 1 = not clear; 2 = somewhat clear; 3 = quite clear; and 4 = highly clear. Following that, the item-level (I-CVI) and scale-level (S-CVI) content validity indices (CVIs) were computed [19]. Two measures of content validity were derived from the CVI: (1) item-level content validity (I-CVI) and (2) scale-level content validity (using the averaging calculation method) (S-CVI). Saefi et al. (2020) recommended keeping items with an I-CVI above 0.800, modifying those between 0.700 and 0.790, and rejecting those below 0.700 [20]. Meanwhile, the S-CVI must be at least 0.800 for content validity to be judged acceptable and for the translated tool to be considered suitable for research [21]. After consulting experts, the wording of certain items was culturally adjusted.

### Study population and settings

#### Phase 2: Exploration and extraction of factors

For the exploration and extraction of factors, a pilot study was conducted on the KOSPEN programme, which is a nationwide initiative to reduce non-communicable diseases from March 2022 to July 2022. The study participants were the communities living in the villages in Sabah where the community programme was held. The study participants were recruited in March 2022. One must be over 18, be able to read and communicate in Malay, have completed at least Standard 5 (Year 5) of primary education, and live in a village or residential area where KOSPEN is organised to be eligible to participate. Sample sizes of 160 to 300 were suggested [22] while sample sizes of 3 to 10 subjects per item are recommended for exploratory factor analysis [23]. Participants were recruited by way of purposive sampling and instruments.

#### Phase 3: Evaluation of the scale

In this phase, an assessment was done on the MIICA programme in Kota Kinabalu, Sabah, to analyse and reaffirm the questionnaire’s final Malay form. It was done from August 2022 to December 2022. The study participants were the marginalized communities living in the villages where the programme was held, and participants were recruited in August 2022. In Phase 3, the inclusion criteria were identical to those used in the Phase 2 study. The sample size followed Hair et al.’s suggestions that the sample size should be 100 or more [24], backed by Comrey and Lee, where 100 was poor, 200 was fair, 300 was good, 500 was very good, and 1000 or more was exceptional [25]. Hence, the sample size was improved to above 200 compared to the sample size in Phase 2.

### Ethical approval

The study was approved by the Medical Research and Ethics Committee (NMRR ID-22-00051-88T (IIR)) Ministry of Health, Malaysia, as well as the ethical committee of University Malaysia Sabah (JKEtika 1/22 (2)). All the participants were above 18 years old and participation was strictly voluntary. Every participant also given their written informed consent form to be involved in the study.

### Data collection and procedures

#### Phase 2: Exploration and extraction of factors

The survey for the pilot study was carried out using an online Google form to contact as many individuals as possible in the Sabah communities where KOSPEN was held. All respondents were made aware that taking part was entirely optional and that the survey’s results would be treated in the strictest confidence. After that, the link to the questionnaire was provided. A statistical analysis was performed on all the responses to the online surveys.

#### Phase 3: Evaluation of the scale

The community-based intervention to improve the measles programme was organised among the marginalised population. The researcher went from house to house to obtain samples from marginalised residents. After explaining the purpose and significance of the study, the researcher then asked for consent. Participation was voluntary, and answers were confidential. Those who agreed would answer the questionnaire.

### Statistical analysis

Information collected in Google Forms and the manual questionnaire from both Phase 2 and 3 of the study was exported to Excel and then analysed using IBM SPSS Statistics 29. Frequency (n) and percentage (%) were used to summarise categorical data for the demographic attributes’ descriptive analysis.

Next, exploratory factor analysis (EFA) was utilised to discover and isolate the components. EFA is a statistical tool used to improve scale reliability by locating inaccurate items that may be removed, establishing dimensionality, and analysing correlations between items and variables in cases where there is insufficient information regarding dimensionality [26]. For this research, EFA was carried out on 12 different variables based on a polychoric correlation matrix. Parallel analysis was used to establish the dimensions, and principal component analysis combined with raw varimax rotation was utilised by Factor software to extract the variables’ components [27]. The polychoric correlation matrix model was used since the questionnaire used ordinal data [28,29]. Subsequently, the sample underwent both Bartlett’s and the Keiser-Meyer-Olkin (KMO) tests to establish whether it was sufficient and appropriate. When conducting factor analysis, a KMO value that is closer to one is preferable; nevertheless, a score larger than 0.500 is normally acceptable, and a score greater than 0.700 is preferable [30]. A significance level of less than 0.050 for Bartlett’s test is acceptable [31]. Positive KMO and Bartlett’s test results suggest that moving forward with the factor analysis is favourable [26,32]. The connection between each component and each survey item is known as factor loading. The association must be supported for the question to be kept. In this investigation, the minimal factor loading was 0.300.

How consistently the results are produced indicates an instrument’s or survey’s reliability. Hence, using SPPS, the scale’s internal consistency was assessed using Cronbach’s alpha coefficient. Internal consistency is considered adequate when Cronbach’s alpha is between 0.800 and 0.700 [33,34]. The survey items typically work well together when there is significant internal consistency. Hence, if someone responds positively to one survey question, they are more likely to respond positively to subsequent ones [33].

Following EFA, the retrieved factors were evaluated using confirmatory factor analysis (CFA) [35]. CFA was used to assess how well the suggested model fit the data. In this work, factor analysis using ordinal data was conducted using the diagonally weighted least squares (DWLS) approach, which obtains higher fit index values with smaller sample sizes [36]. The R software’s lavaan and semTools packages were used for the research. Model fit indicators in CFA are divided into three broad categories: (1) absolute fit: goodness-of-fit index (GFI), chi-square, and standardised root mean square residual (SRMR); (2) parsimonious fit: PCFI, PNFI, AGFI, and AIC; and (3) comparative fit: comparative fit index (CFI), incremental fit index (IFI), regression fit index (RFI), normal fit index (NFI), and TLI [37].

This study’s criteria for determining the goodness of fit were GFI, CFI, RMSEA, SRMR, TLI, and chi-square. The chi-square checks how well the sample and fitted covariance matrices fit together and how much they differ. Nonetheless, sample sizes matter, and the p-value should be above 0.050. (i.e., the hypothesis of a perfect fit cannot be rejected). Following this, the Standardised Root Mean Square Residual (SRMR) is the value that should be less than 0.080 and is the square root of the difference between the residuals of the sample covariance matrix and the hypothesised model. The Root Mean Square Error of Approximation, also known as RMSEA, is an index that encompasses parsimony. A satisfactory fit is indicated by values that are closer to 0.000. An unfit model will have a larger RMSEA score [38], while a fit model should have a score of less than 0.080 or 0.050. The Comparative Fit Index (CFI), which compares the fit of a target model to the fit of an independent or null model, should be more than 0.900. Finally, TLI should be greater than 0.900 or greater than 0.950 [39], indicating that the model of interest improves fit. The value regarded as a good fit for the GFI and AGFI was > 0.900 [40]. After looking at the factor loadings, the composite reliability was utilised to verify the factors’ reliability.

## Results

### Sample characteristics

Phase 2 of the investigation involved 170 samples, while Phase 3 involved 235 samples. **Table 1** displays the sample characteristics for both phases of the study. The cohorts in Phases 2 and 3 were similar in that all participants lived in locations where a community-based intervention was conducted with the help of community volunteers.

**Table 1:** Participants’ sociodemographic characteristics.

Characteristics	Phase 2: Exploration and Extraction of Factors, n (%)	Phase 3: Evaluation of Scale, n (%)
Gender Male Female	48 (37.8%) 79 (62.2%)	25 (10.6%) 210 (89.4%)
Age 18–24 25–54 55–64 > 65	4 (2.4%) 107 (62.9%) 58 (34.1%) 1 (0.6%)	63 (26.8%) 164 (69.8%) 6 (2.6%) 2 (0.9%)
Education level No school certificates Primary school Secondary school University	0 0 149 (87.6%) 21 (12.4%)	19 (8.1%) 113 (48.1%) 103 (43.8%) 0
Employment status Unemployed Employed Retired	38 (22.4%) 123 (72.4%) 9 (5.3%)	210 (89.4%) 25 (10.6%) 0
Area of Residency Urban Rural	23 (15.6%) 147 (86.5%)	235 (100%) 0

### Translation

The forward-backward translation was reviewed to ensure the Malay translation was as accurate as the original and did not lose any validity or reliability. All items (n = 12) in all four constructs were kept after comprehensive discussion and review with expert committees.

### Face and content validity

The content validity index was utilised to evaluate the Malay translation of the AIM-IAM-FIM questionnaire. The result of the item-level CVI ranges showed that one item was 0.667, two items were 0.778, six items were 0.889, and three items were 1.000 **(Table 2)**. Although the lowest score for item 1 was 0.667, the item was maintained after discussion with the experts since it is meaningful. The total S-CVI score calculated from all three constructs was 0.880, which is considered acceptable for content validity. Nine experts confirmed the items’ face validity, and upon evaluation, several adjustments were made to improve simplicity and clarity.

**Table 2:** Content validity analysis result.

No.	Item	I-CVI	Kappa	Decision
**Acceptability (S-CVI = 0.833)**				
Item1	MIICA/ KOSPEN programme meets my approval *“‘Program MIICA / KOSPEN memenuhi persetujuan saya”*	0.667	0.601	Item retained
Item2	MIICA/ KOSPEN programme is appealing *“‘Program MIICA/ KOSPEN menarik minat saya”*	0.889	0.887	Item retained
Item3	I like the MIICA/ KOSPEN programme *“Saya suka program MIICA/ KOSPEN*”	0.889	0.887	Item retained
Item4	I welcome the MIICA/ KOSPEN programme *“Saya menyambut program MIICA/ KOSPEN dengan baik”*	0.889	0.887	Item retained
**Appropriateness (S-CVI = 0.972)**				
Item5	MIICA/ KOSPEN seems fitting *“‘Program MIICA/ KOSPEN kelihatan bertepatan dengan objektif”*	0.889	0.887	Item retained but rephrased
Item6	MIICA/ KOSPEN seems suitable *“Program MIICA/ KOSPEN kelihatan bersesuaian dengan masyarakat”*	1.000	1.000	Item retained but rephrased
Item7	MIICA/ KOSPEN seems applicable *“Program MIICA/ KOSPEN kelihatan boleh digunapakai oleh masyarakat”*	1.000	1.000	Item retained
Item8	MIICA/ KOSPEN seems like a good match *“Program MIICA/ KOSPEN kelihatan berpadanan baik dengan program imunisasi/ kesihatan sedia ada”*	1.000	1.000	Item retained
**Feasibility (S-CVI = 0.833)**				
Item9	MIICA/ KOSPEN seems implementable *“‘Program MIICA/KOSPEN kelihatan boleh dilaksanakan dalam masyarakat”*	0.778	0.761	Item retained but rephrased
Item10	MIICA/ KOSPEN seems possible *“‘Program MIICA/ KOSPEN kelihatan boleh dijalankan oleh masyarakat”*	0.778	0.761	Item retained but rephrased
Item11	MIICA/ KOSPEN seems doable *“Program MIICA/ KOSPEN kelihatan boleh dilakukan oleh komuniti dan sukarelawan”*	0.889	0.887	Item retained
Item12	MIICA/ KOSPEN seems easy to use *“‘Program MIICA/ KOSPEN kelihatan mudah untuk dijalankan”*	0.889	0.887	Item retained but rephrased

S-CVI: Scale Content Validity Index; I-CVI: Item Content Validity Index.

After considering their usage in Malaysian scenarios, several words and phrases were added to define the correct meaning. These include adding the word *“program”* after the acronym MIICA for every item; adding the word *“objektif”* for objective for item 5; *“masyarakat”* for community for items 5, 6, 9, and 10; *“program imunisasi”* for immunisation programme for item 8; and *“sukarelawan”* for volunteer at the end of the sentence for item 11 to help readers comprehend the questions.

### Exploratory factor analysis

This study used the three constructs of applicability, appropriateness, and feasibility to determine the structure of the 12-item AIM-IAM-FIM Questionnaire.

### Preliminary three-factor structure

An initial analysis was performed to determine the eigenvalues of each item in the data. However, a 69.1% covariance was identified. The analysis removes item 2 *(Program MIICA menarik minat saya)*, which correlates 0.973 with item 3 *(Saya suka program MIICA)*. Upon rerunning the analysis, the result showed the presence of 27.6% covariance. The KMO test result was 0.930, with Bartlett’s sphericity result being significant (1893.9, df = 55, p = 0.000). However, the number of factors determined was only one. Several elements with high correlations (> 0.900) were eliminated to improve the study further. The following items were removed: item 4 *(Saya menyambut program MIICA dengan baik);* item 5 *(Program MIICA kelihatan bertepatan dengan objektif);* item *7 (Program MIICA kelihatan boleh digunapakai oleh masyarakat);* and item 12 *(Program MIICA kelihatan mudah untuk dijalankan)*. Comprehensibility was the primary factor when comparing and selecting the items to be kept. The final computation is maintained in one dimension.

### Final single factor structure

Following the removal of five items that had significant correlations with one another, the final one-dimensional structure in this investigation consisted of seven items. This group of items produced a KMO test result of 0.928, indicating a good sample size. Bartlett’s sphericity test was also significant (1909.2, df = 21, p = 0.000). According to the findings presented in **Table 3**, item one of this seven-item structure was responsible for 81.5% of the variance in the relationship pattern that existed between the items. Item 2 explained 6.4% of the variance, item 3 explained 5.2%, item 4 explained 3.8%, item 5 explained 2.8%, item 6 explained 0.05%, and item 7 explained 0.001%. The correlation between the items is shown in **Table 4**.

**Table 3:** Eigenvalue and total variances explained for the final single structure.

Item	Eigenvalue	Proportion of Variance	Cumulative Proportion of Variance
1	5.708	0.815	0.815
2	0.453	0.064	
3	0.370	0.053	
4	0.267	0.038	
5	0.198	0.028	
6	0.003	0.000	
7	0.000	0.000	

Extraction method: Principal Component Analysis.

**Table 4:** Correlation matrix.

	Item 1	Item 3	Item 6	Item 8	Item 9	Item 10	Item 11
Item 1	1.000						
Item 3	0.697	1.000					
Item 6	0.900	0.766	1.000				
Item 8	0.698	0.715	0.808	1.000			
Item 9	0.690	0.676	0.944	0.751	1.000		
Item 10	0.699	0.697	0.905	0.923	0.938	1.000	
Item 11	0.708	0.760	0.796	0.762	0.809	0.897	1.000

Extraction method: Principal Component Analysis; Rotation method: Raw Varimax.

### Reliability

Cronbach’s alpha was utilised to evaluate the degree to which the structure exhibited reliable internal consistency. The overall Cronbach’s Alpha value for the scale was 0.938, and removing the item did not result in appreciable shifts in the values of the other variables. Because the value of Cronbach’s alpha was greater than or equal to 0.700, it was deemed satisfactory.

### Confirmatory factor analysis

The confirmatory factor analysis result showed chi-square = 34.544, with df = 14, and a p-value of 0.002; CFI = 0.999; TLI = 0.998; RMSEA = 0.079; and SRMR = 0.030 **(Table 5).**

**Table 5:** Fit indices of the models.

Model	Items	X^2^(df)	P value	SRMR	RMSEA	CFI	TLI
1	7 items	34.55 (14)	0.002	0.030	0.079	0.999	0.998
2	6 items (Item 9 removed)	26.995 (9)	0.001	0.031	0.092	0.999	0.998

SRMR = Standardised Root Mean Square Residual; RMSEA = Root Mean Square Error of Approximation; CFI = Comparative Fit Index; TLI = Tucker-Lewis Index.

**Fig 2:**
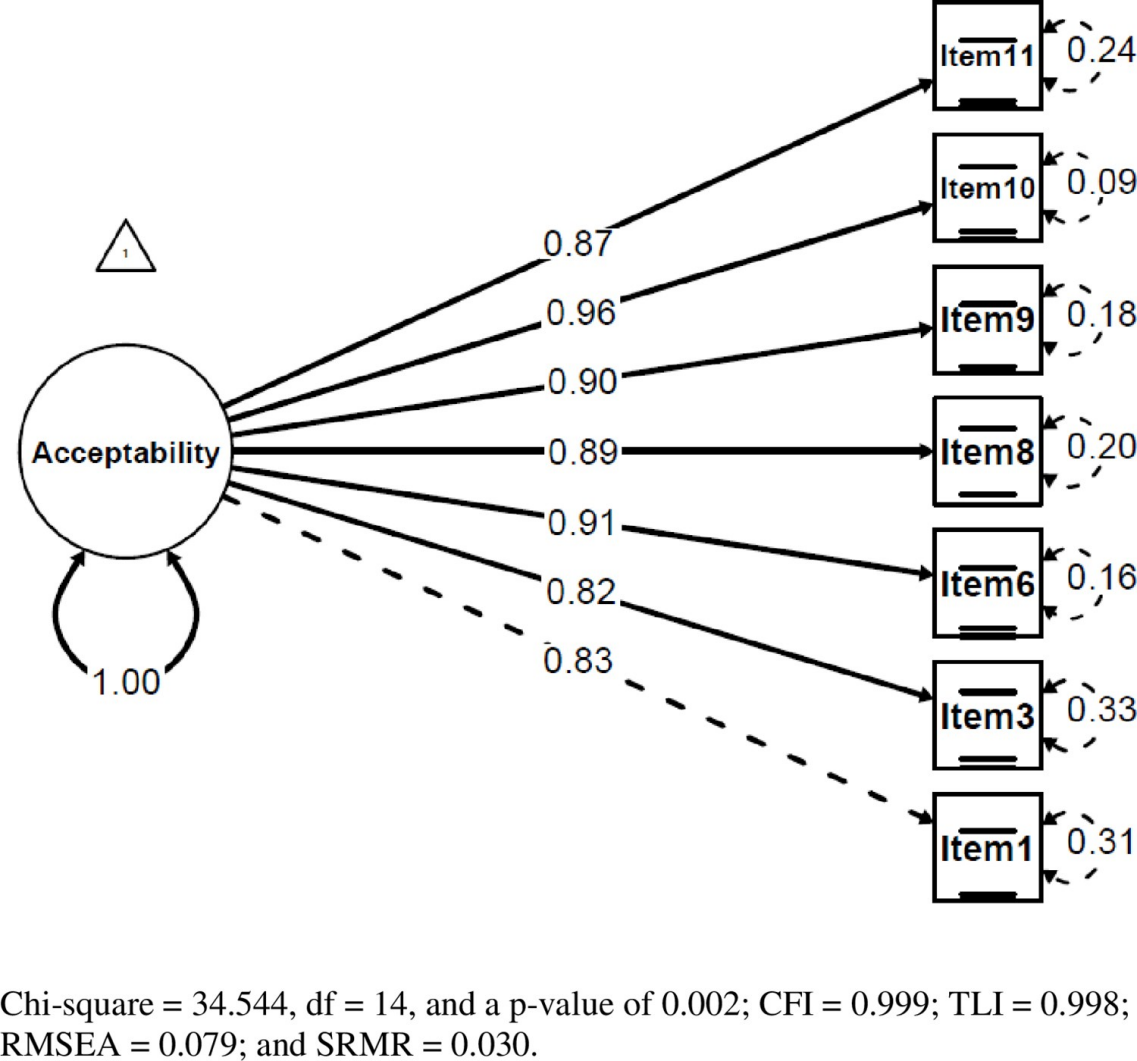
The final structure of the model.

Items with high correlation could be eliminated from the single-factor form to improve the structure’s fit further. From Table 3, a high correlation was seen between item 6 *(Program MIICA kelihatan bersesuaian dengan masyarakat)* and item 9 *(Program MIICA kelihatan boleh dilaksanakan dalam masyarakat)*, which was 0.944. As a result, CFA was evaluated by deleting item 9 because item 6 was more comprehensible than item 9. The result was similar, with seven items in the single factor structure compared to 6 items with chi-square = 26.995, df = 9, and a p-value of 0.001; CFI = 0.999; TLI = 0.999; and SRMR = 0.031. However, RMSEA = 0.092, which was > 0.08, indicates that having all 7 items in the single factor structure was better than having 6 items. Hence, all the values reached a satisfactory degree of goodness of fit. In addition, the CFA findings were plotted, as shown in **Fig 2**. All items had factor loadings greater than 0.700, indicating reliable items in the structure.

## Discussion

The objective of this study was to translate and assess the psychometric features of the AIM-IAM-FIM questionnaire in the Malay language. This research is significant because it provides programme managers with tools to determine how successfully a programme has been implemented, particularly when there are limited tools for evaluating how well a health initiative is being implemented in Malaysia. Numerous community-based programmes are being established in Malaysia, not just in health. Therefore, having validated and reliable implementation outcome measures for future applications is beneficial. Several studies have already utilised these questionnaires to examine the outcomes of programmes organised in their nation [13,41,42].

The Malay version of the AIM-IAM-FIM questionnaire’s validity and reliability were verified using precise techniques based on psychometric standards. Qualified translators and expert panels validated the tool, attesting to the instrument’s conformity to the original English edition’s subject matter, concept, standard, and contextual criteria. As a result, this study exhibited strong face and content validity, indicating that the items after translation were simple to comprehend.

However, when comparing the sociodemographic characteristics of the participants between the two community based programmes (KOSPEN and MIICA) used in this study, it was noted that there were differences in the gender, education level, employment status and area of residency characteristics. These were due to the different localities where the programmes were organised. Both programmes were done in the district of Kota Kinabalu. MIICA programme was specifically done on the Gaya Island which was 10 minutes away by boat from the city centre while KOSPEN was done on the villages on land within the district. All the participants were approached during the day. As such, most of participants on the island were women since the husband went out for work at those times and majprity were unemployed and only have the standard education level up to secondary school. Nevertheless, the questionnaire was simple, easy to understand and the participants could answered them without assistance. Thus, their sociodemographic differences did not influenced the way they understood or answered the questionnaire as Malay is the main language of all the participants in the community within the district of Kota Kinabalu. Importance was placed on the similar way both community-based programmes was done to evaluate the implementation outcome at the end of both initiatives.

Nevertheless, the exploratory factor analysis revealed a seven-item, one-dimensional survey. This result differs from the original and another study conducted in Germany with three constructs [7]. Regardless, the study’s findings on internal consistency confirm the accuracy and validity of the implementation outcome measures for the community-based intervention. The overall single-factor structure of the result demonstrated strong internal consistency with Cronbach’s alpha of 0.940, exceeding the minimally acceptable level of 0.700 and being consistent with the original study [7]. Following that, confirmatory factor analysis demonstrated that the structure adequately matched the data and provided significant support for the factorial validity of the scale. The robust fit indices, which were χ2 (p-value > 0.050), CFI and TLI of ≥ 0.950, and RMSEA and SRMR of ≤ 0.080, were used to decide whether to approve the model fit. The seven questionnaire items were retained, considering the significance and applicability of each item and domain.

Thus, the final Malay version of the survey was shorter with adequate fit indices. Yet, comparable to this study, the questionnaire that was verified among German school teachers yielded 11 questions and a shorter questionnaire [42]. Although there is still limited research that has evaluated this questionnaire’s psychometric properties, the decrease in the dimensions and the items may be due to cultural, demographic, and geographical variations in the studied population. Regardless, it is much easier and more practical to complete the questionnaire when it is shorter since the participants in this study came from a wide range of backgrounds in terms of age, education, and socioeconomic status. A shorter questionnaire ensures a better response rate, completion rate, and accuracy of the data [8].

The research’s limitations include having a high correlation between the items, which highlights that conceptually different implementation outcomes are hard to tell apart in practice. Based on this current study’s findings, the community in Sabah considered practicability, appropriateness, and acceptability essentially the same. Further studies can be done in different states in Malaysia, which may have different cultures and backgrounds than those in this current study, to test the outcome measures further.

## Conclusion

The translated and validated Malay versions of the AIM-IAM-FIM questionnaire had 7 items and primarily assessed the acceptability of the community-based intervention programme based on the results from the exploratory and confirmatory factor analysis. This study adds to the literature on the validation of this questionnaire that had been done in other countries in different languages. The Malay version of this tool is a valid and reliable instrument that can be practically utilised to evaluate the implementation outcomes of any community-based programmes in any discipline in Asian countries who had citizens who conversed in Malay language. Future studies can further explore and test the psychometric criteria of this questionnaire in different context and environment according to their own countries. When the questionnaire is used, the findings could then guide future policy in regards to the acceptance of the community on the community-based programme implemented on them.

## Supporting information

S1 Dataset For EFA. https://doi.org/10.7910/DVN/EKF8BH. (CSV)

S1 Dataset For CFA. https://doi.org/10.7910/DVN/FZFVRY. (CSV)

S1 File AIM-IAM-FIM questionnaire. (PDF)
