# Supplementary material for: Translation, Cross-Cultural Adaptation to Malay, and psychometric evaluation of the AIM-IAM-FIM questionnaire: Measuring the implementation outcome of a community-based intervention programme
Source: PLoS One. 2023 Nov 16;18(11):e0294238. doi: 10.1371/journal.pone.0294238 (PMC10653410; doi:10.1371/journal.pone.0294238)
Supplement: S1 File — (PDF) [file pone.0294238.s003.pdf]

AIM-IAM-FIM questionnaire (Malay Version)

KEBOLEHTERIMAAN INTERVENSI

Sila jawab soalan dengan mengisikan tempat kosong atau tandakan (√) pada jawapan yang paling sesuai berdasarkan penerimaan anda terhadap Program Intervensi Imunisasi Campak melibatkan Tindakan Komuniti atau *Measles Immunization Intervention involving Community Action* (MIICA) *programme*.

Kebolehterimaan Langkah Intervensi (*Acceptability of Intervention Measure* atau *AIM*)

	Sangat tidak setuju	Tidak setuju	Neutral	Setuju	Sangat setuju
1. Program MIICA memenuhi persetujuan saya	①	②	③	④	⑤
2. Program MIICA menarik minat saya	①	②	③	④	⑤
3. Saya suka program MIICA	①	②	③	④	⑤
4. Saya menyambut program MIICA dengan baik	①	②	③	④	⑤

Kesesuaian Langkah Intervensi (*Intervention Appropriateness Measure* atau *IAM*)

	Sangat tidak setuju	Tidak setuju	Neutral	Setuju	Sangat setuju
1. Program MIICA kelihatan bertepatan dengan objektif	①	②	③	④	⑤
2. Program MIICA kelihatan bersesuaian dengan masyarakat	①	②	③	④	⑤
3. Program MIICA kelihatan boleh digunapakai oleh masyarakat	①	②	③	④	⑤
4. Program MIICA kelihatan berpadanan baik dengan progam imunisasi sedia ada	①	②	③	④	⑤

Kebolehlaksanaan Langkah Intervensi Feasibility of Intervention Measure atau FIM)

	Sangat tidak setuju	Tidak setuju	Neutral	Setuju	Sangat setuju
1. Program MIICA kelihatan boleh dilaksanakan dalam masyarakat	①	②	③	④	⑤
2.Program MIICA kelihatan boleh dijalankan oleh masyarakat	①	②	③	④	⑤
3. Program MIICA kelihatan boleh dilakukan oleh komuniti dan sukarelawan	①	②	③	④	⑤
4. Program MIICA kelihatan mudah untuk dijalankan	①	②	③	④	⑤

AIM-IAM-FIM questionnaire (English Version)

INTERVENTION ACCEPTABILITY

Please answer the questions by filling in the blanks or ticking (√) at the most appropriate answer based on your acceptance on the Measles Immunization Intervention involving Community Action (MIICA).

Acceptability of Intervention Measure (AIM)

	Completely disagree	Disagree	Neither agree nor disagree	Agree	Completely agree
1. MIICA meets my approval.	①	②	③	④	⑤
2. MIICA is appealing to me.	①	②	③	④	⑤
3. I like MIICA	①	②	③	④	⑤
4. I welcome MIICA	①	②	③	④	⑤

Intervention Appropriateness Measure (IAM)

	Completely disagree	Disagree	Neither agree nor disagree	Agree	Completely agree
1. MIICA seems fitting.	①	②	③	④	⑤
2. MIICA seems suitable.	①	②	③	④	⑤
3. MIICA seems applicable.	①	②	③	④	⑤
4. MIICA seems like a good match.	①	②	③	④	⑤

Feasibility of Intervention Measure (FIM)

	Completely disagree	Disagree	Neither agree nor disagree	Agree	Completely agree
1. MIICA seems implementable.	①	②	③	④	⑤
2.MIICA seems possible.	①	②	③	④	⑤
3. MIICA seems doable.	①	②	③	④	⑤
4. MIICA seems easy to use.	①	②	③	④	⑤
